# More streamlined and targeted. A comparative analysis of the 7th and 8th Environment Action Programmes guiding European environmental policy

**DOI:** 10.1016/j.heliyon.2023.e19212

**Published:** 2023-08-22

**Authors:** Lavinia C. Pindaru, Andreea Nita, Iulian M. Niculae, Steluta Manolache, Laurentiu Rozylowicz

**Affiliations:** a Centre for Environmental Research, University of Bucharest, Bucharest, Romania; b Doctoral School in Geography Simion Mehedinti - Nature and Sustainable Development, University of Bucharest, Bucharest, Romania

**Keywords:** Institutional Grammar Tool, Network analysis, Environmental governance, European Union policies, European Green Deal, 2030 Agenda for Sustainable Development

## Abstract

Environment Action Programmes (EAP's) are the most important documents defining the environmental policies within the European Union. Their implementation, over the previous 50 years, represented a significant advance in raising eco-friendly awareness and suggesting solutions for environmental problems in the European Union. In this paper, we used Institutional Grammar Tool and network analysis to identify the evolution of EU EAP's by investigating the most recent two programmes (7th Environment Action Programme and 8th Environment Action Programme), particularly in priority objectives, institutional statements, enforcement perspectives, and projected participation of stakeholders. We found that the EU's 8th Environment Action Programme (2021–2030) is further streamlined and target oriented as compared to 7th Environment Action Programme. Furthermore, institutional statements included in the 8th EAP will be implemented predominantly at the levels of European Union and European Commission. On the contrary, in the 7th EAP, the number of institutions, frameworks, and stakeholders is higher and often regional and local (e.g., European Union, Environment Action Programme, European Environment Agency, European Commission, European Parliament, Convention on Biological Diversity, regional authorities, local authorities). The close links of the 8th EAP targets with the 2030 Agenda for Sustainable Development and the European Green Deal represent an important step towards a greater applicability of environmental policies in the European Union. Our study reveals that comparative analysis of legal documents using Institutional Grammar Tool and network analysis can assist policymakers in assessing the drafting of legal environmental documents and obtain indispensable information about the changes to improve environmental policies.

## 1 Introduction

Governance represents the process by which societal actors exercise their authority, formulate decisions, and propose actions [1]. Governance includes the totality of institutions, structures, and processes related to decision making. This also applies to environmental governance [2], which is currently focused on analysing the networks of actors and communities that develop and implement environmental policies, including understanding how environmental decisions are created [3,4]. Analysing environmental governance may help develop and implement more efficient environmental policies [[5], [6], [7], [8]].

Environmental policies represent measures to strengthen the relationship between the natural environment and humans in a mutually beneficial manner [[9], [10], [11], [12]]. Environmental policies represent not only a set of principles that guide stakeholders in making the best decisions regarding the well-being of the environment and the public [[13], [14], [15], [16]] but also norms containing binding rules for various formal and informal institutions [3,17,18].

The history of environmental policy at the EU level can be divided into four periods, each leading to the consolidation of sustainability of EU countries [[19], [20], [21]]. The start of the first period is represented by the signing, in 1957, of the Treaty of Rome [22,23]. The start of the second period is represented by the introduction of the Environment Action Programmes in 1973 (the first Environment Action Programme was in force between 1973 and 1976) and represents the first attempt to recognise the importance of the environment in the well-being of the EU population [22]. The start of the third period is represented by the signing of the Single European Act in 1987, when the new provisions regarding environmental policies created many legal ambiguities. The last key period started with the signing of the Maastricht Treaty in 1992 [23], when a specific legal basis for environmental health as a European problem was introduced [24].

EU Environment Action Programmes (EAPs) represent strategic documents reflecting key environmental issues in the EU. The European Commission adopted the First Environment Action Programme in 1973, following the recommendations of the UN Stockholm Conference (1972). The first EAP outlined potential legislative proposals and environmental policy goals at the EU level [25]. From 1973 to 2022, the EU promoted eight EAPs. Their focus demonstrated progress, covering more complex environmental themes, e.g., environmental policy making, maintaining the overall quality of life, and sustainable development. Thus, EAPs offered a robust legislative basis and significantly guided environmental legislation [26].

Due to the importance of environmental policy for the well-being of the environment and the public, this issue has been evaluated and extensively analysed: for example, employing institutional analysis of policy documents and institutions [27], e.g., using institutional grammar tool, discourse analysis, content analysis, and text mining. Since there are gaps in the understanding of environmental policies and their applicability, using the institutional grammar tool, it was possible to extract precise information regarding the major differences between the legislative documents with applicability at the level of the EU member states.

Institutional Grammar Tool (IGT) emerged as a useful policy analysis method due to its superior accuracy in extracting relevant information from the content of policy documents, being able to classify the types of statements (strategies, norms, rules), the types of actions (permitted, mandatory, prohibited), as well as the types of conditions (spatial, temporary, procedural) [28]. IGT is underused compared to its potential due to the lack of recommendations for using documents in languages other than English and the lengthy processing time [29,30]. However, this tool can assists in translating legislative concepts with the goal of providing a much clearer framework of the content [31]. Additionally, IGT is an analytical tool that classifies and organises the content of policies [32] and might be used to identify the main unresolved challenges for implementation and simulate the applicability of legal norms [33]. Furthermore, the database obtained by applying IGT may be used in network analysis, helping to visualise representative connections in the document. Network analysis supports the identification of any connections between institutional statements and attributes of statements. Network analysis explores the existing connections and the dynamics and relationships that influence stakeholders nominated in the analysed documents [34,35].

Given the importance of Environment Action Programmes for the well-being of the environment and people in the EU, this study aims to assess the evolution of environmental policies promoted by the European Union by analysing the rules, norms, and strategies included in the most recent EU Environment Action Programmes and offers insights for policy makers to improve these legal documents. Specifically, the objectives of this article are: (1) to document the evolution of the last two EAPs thematic priorities objectives as an indicator of long-term environmental policy focus, (2) to understand the changes in the structure of EU environmental governance by classifying EAPs statements into rules, norms, or strategies using the Institutional Grammar Tool framework, and (3) analysing EAPs enforcement perspectives using the deontic component of institutional statements (deontic verbs) and network analysis using the attribute component of institutional statements to identify links between actors (institutions, frameworks, stakeholders) and types of statements.

## 2 Methods

### 2.1 Environment Action Programmes

To understand the evolution of EU EAPs, we analysed two documents out of eight issued to date, namely the 7th Environment Action Programme covering the period 2013–2020 and the 8th Environment Action Programme covering the period 2021–2030. The two EAPs were retrieved from the official repository of the European Commission (https://eur-lex.europa.eu/legal-content/EN/TXT/?uri=CELEX:32013D1386 for the 7th EAP and https://eur-lex.europa.eu/legal-content/EN/TXT/?uri=CELEX:32022D0591 for the 8th EAP). We selected the two programmes because they represent the most recent legislative documents, having the potential to highlight changes in environmental policies.

The 7th Environment Action Programme, "Living well, within the boundaries of our planet", was adopted by the European Parliament and the Council of the European Union in 2013. This document entered into force in 2014 and set EU environmental objectives to be met by 2020 [36]. The 7th EAP includes objectives that address key negative environmental trends in the EU: biodiversity, habitats and soils which were declining; water and air qualities were detrimental in several regions of Europe; and the public was significantly exposed to harmful substances that affected their health and overall well-being [37,38]. The 7th EAP includes nine priority objectives divided into three thematic, four enabling framework, and two horizontal objectives [38].

The 8th Environment Action Programme entered into force in May 2022 and will guide the EU's environmental policy until 2030. It reiterates the long-term vision of the EU (2050 time horizon), namely, to live well within the planet's resource limits and move towards an economy as sustainable as possible, taking into account the objective of preserving a healthy ecosystem. The 8th EAP emphasises the full involvement of stakeholders at all levels of governance to ensure that the environmental policy objectives will be achieved [39]. The 8th EAP includes six priority objectives to be met by 2030 and establishes a list of indicators to monitor the achievement of the proposed objectives [37].

### 2.2 Coding with institutional grammar tool

Institutional Grammar Tool is an effective method for analysing legal documents and their evolution in terms of structure and content, first developed and applied by Crawford and Ostrom in 1995 [29]. The main objective of the IGT is to systematically examine the content of legal documents as regards statement structures. It helps to define the behaviours of institutions and the actors involved in policy implementation and policy making [40]. IGT also separates specific components by analysing linguistic constructions [27] and determining whether an action is compliant or noncompliant, permitted, mandatory, or prohibited [41]. IGT is applied in two stages, i.e., identification of institutional statements (linguistic constructions that describe opportunities and constraints, which can influence actors and their actions) and interpretation of their syntax, by decomposing each statement into 6 components (ABDICO): attribute (*A*) – an individual or organisation to whom the institutional statement relates; object (*B*) – the inanimate or animate part of an institutional statement that is the receiver of the action described in the aim; deontic (*D*) – the prescriptive element of a legislation that specifies what is idealistically allowed (e.g., may, should, can, could), required (e.g., must, shall, required, need), or prohibited (e.g., must not, shall not, can not, may not); aim (*I*) – the goal or action of the statement that the Deontic refers; condition (*C*) – the boundaries of an action's execution in terms of space, time, and/or procedure; and or else (*O*) – the punitive sanction imposed for breaking a rule [28,30,[42], [43], [44]].

The components of each statement indicate the type of statement, in our case, AIC, ABIC, ADIC, and ABDIC (Table 1). AIC/ABIC statements type (components: Attribute, oBject, aIm and Condition) are considered strategies, i.e., a routine plan to implement the policy [41]. An AIC statement represents a simple strategy (AIC simple strategy), while ABIC statement indicates a complex strategy (ABIC complex strategy). Both types of strategies include an Attribute, an aIm, and a Condition. However, an ABIC complex strategy includes an oBject, i.e., the receiver of the action described in aIm. ADIC/ABDIC statement types are considered as norms, i.e., recommendations for actions that are to be carried out, and it is assigned [41]. Similarly to strategies, the ADIC statement represents a simple norm (ADIC simple norms), while the ABDIC statement indicates complex norms (ABDIC complex norms). Both types of norms include an Attribute, a Deontic, an aIm, and a Condition. An ABDIC complex norm also includes an oBject, i.e., the receiver of the action described in the aim, thus being more rigorous [41]. Other types of statements are the rules, a collective prescription for policy and the sanction for violating them. These are assigned to ADICO/ABDICO categories (components: Attribute, oBject, Deontic, aIm, Condition, and Or else) [43,45]. Rules-type statements were not identified in the analysed documents. As an illustration of coding, the statement "The Union and its Member States should also reflect as soon as possible on how soil quality issues could be addressed … " from 7th EAP includes an Attribute (The Union and its Member States), an oBject (soil quality issues), a Deontic (should), an aIm (reflect) and a Condition (as soon as possible), therefore is an ABDIC type of statement (complex norm). Interpretation of the two EAP's statements using the Institutional Grammar Codebook [43] was performed over six months by the first author of this paper (the 7th EAP is 30 pages long, while the 8th EAP is 15 pages long).

Using these types of statements, we analysed the enforcement perspectives of each priority objective and the environmental area covered. Depending on the deontic included in each statement, we divided the actions into not regulated (deontic is absent), permitted (a statement that describes an action that can be implemented), mandatory (a statement that presents an order), and prohibited (a statement that describes the action that the attribute must not do). Then, we assigned each statement to a priority objective and one or several environmental domains (water, air, soil, biodiversity, climate, chemical substances, waste and cycling, human health, energy, resource management, economy, and others). The environmental domain was assigned to each statement using the keywords detailed in Table 2. Due to the complexity of EAPs, an institutional statement can cover more than one environmental domain, especially for those with two or more conditions in their structure.

**Table 1:** Examples of IGT coding for AIC, ADIC, ABIC and ABDIC institutional statements.

Statements type		Acronym	Statement component				
			Attribute	Object	Deontic	Aim	Condition
Simple statements	Strategy	AIC	the Union	–	–	will engage	proactively
	Norm	ADIC	The Union and its Member States	–	shall	contribute	to a high level of environmental protection
Complex statements	Strategy	ABIC	The Union and its Member States	soil quality issues	–	reflect	as soon as possible
	Norm	ABDIC	The Union	products	should	be	sustainably sourced

**Table 2:** Components of environmental domains identified in the 7th and 8th Environment Action Programmes.

Domains	Definition
Water	Clean water, quality, urban wastewater, drinking water, bathing water, marine areas, water quality, rivers, coastal water
Air	Clean air, gas emissions, air quality, carbon sequestration, air pollution, reduce emissions, ozone-depleting substances, atmospheric pollution
Soil	Pesticides, nutrients, biocides, fertilisers, soil quality, acidification
Biodiversity	Environmental protection, ecosystem services, protection of nature, forests, trees, species, fishing, animal, biodiversity loss
Climate	Climate change, greenhouse gas, gas emissions, temperature increase, carbon footprints
Chemical substances	Hazardous substances, carbon sequestration, CO_2_ emissions, nutrients, nitrogen, phosphorus
Waste and recycling	Recycling activities, waste management, prevention, recycling, disposal, food waste, hazardous waste
Human health	Noise pollution, chemical exposure and toxicity, light pollution, green spaces
Energy	Friendly technologies, energy efficiency, marine biotechnology, renewable energy, greener technologies
Resourcemanagement	Resource efficiency, land management, water management, environmental management systems, sustainable management
Land use	Land degradation, farmland, pressures on land, agroforestry, land-use, agricultural areas, traditional practices
Economy	Industry, agriculture, transport, tourism, circular economy, land fragmentation, aquaculture, investment, market, green economy, nanomaterials, eco-innovation
Other	Government, public participation, policy framework, research, legislation, agreements

### 2.3 Network analysis of stakeholders and statements types

Network analysis is a research tool that can illustrate network structures and diagnose the collaboration process [46]. To better understand the differences between the two EAPs, for each EAP, we constructed a bipartite network connecting stakeholders of each institutional statement (attribute in IGT syntax) with the type of institutional statement resulted from IGT coding (that is, AIC simple strategy, ABIC complex strategy, ADIC simple norm, ABIC complex norm). A bipartite network has two *node* partitions [47,48], which in our case, are the stakeholders to whom the institutional statement relates and the type of the statement. The link (*edges*) between the stakeholders and a type of statement is established when the respective actors are mentioned in the statement. To observe if there are significant differences between the two EAPs network structures [49], for each network, we calculated the following network-level metrics: network density (the number of connections in the network divided by maximum connections that could be formed in the network; indicate which network is more compact or to what degree the stakeholder participates in each type of actions), diameter (the length of the longest geodesic between the two most distant indirectly connected stakeholders/types of statements in the network), transitivity (a measure of the tendency of the stakeholders/types of statements to cluster together) and fragmentation (proportion of stakeholders/types of statements that cannot reach each other, i.e., the stakeholders may act in isolation). We also calculated the normalised degree centrality (the number of statements types having at least a stakeholder in common, divided by the maximum possible degree) to identify which of the nodes are most connected in the EAPs networks. This approach allowed us to highlight key attributes and statements with high centrality [50]. Network representations and analyses were performed using UCINET and NetDraw software [51].

## 3 Results

### 3.1 Changes of EU environmental priorities

The priority objectives of the 7th and 8th EAPs have changed in terms of the number and subject of environmental themes covered. The 7th EAP lists nine priority objectives (Table 3), while the 8th EAP has only six (Table 4). 7th EAP includes priorities related to natural capital, transformation of economy, health and human well-being, law enforcement, cities, integration of environment into other policy areas and increasing the international support of the EU. On the contrary, the 8th EAP shows a more practical approach by including priorities related to biodiversity, climate change, and zero-emission economy. Furthermore, the formulation of objectives is more imperative in the 8th EAP compared to the 7th EAP. For example, priority objectives in the 7th EAP include verbs such as protect, conserve, enhance, turn, safeguard, increase, and ensuring, while the 8th EAP includes verbs such as reduce, adapt, achieve, protect, and restore.

**Table 3:** The priority objectives of the 7th Environment Action Programme.

No	Priority objectives	Theme
1	to protect, conserve and enhance the Union's natural capital	Natural capital
2	to turn the Union into a resource-efficient, green, and competitive low-carbon economy	Economy
3	to safeguard the Union's citizens from environment-related pressures and risks to health and well-being	Human health and well-being
4	to increase the benefits of Union environmental legislation by improving its implementation	Law enforcement
5	to develop environmental knowledge and expand the policy database	Knowledge
6	ensuring investment in environmental and climate policy and justifying the environmental costs of any activities related to society	Economy
7	better integration of environmental considerations into other policy areas and ensuring coherence when formulating new policies	Integration into other policy areas
8	to increase the sustainability of Union cities	Sustainable cities
9	to support the Union for a more effective approach to environmental and climate challenges at international level	International dimension

**Table 4:** The priority objectives of the 8th Environment Action Programme.

No	Priority objectives	Theme
1	to reduce greenhouse gas emissions	Greenhouse
2	to adapt to climate change	Climate change
3	to achieve a regenerative growth model	Growth model
4	to reduce pollution to zero	Zero pollution
5	to protect and restore biodiversity	Biodiversity
6	to reduce the main environmental and climate consequences of production and consumption	Sustainable economy

### 3.2 Evaluation of institutional statements in the analysed EAPs

After coding each statement in the two EAPs following the Institutional Grammar Codebook [43], we found significant differences in the total number and types of institutional statements. The two EAPs include 313 institutional statements (259 in 7th EAP and 54 in 8th EAP), of which 126 are strategies (statements that describe the behaviour of an actor, which does not include any constraint or internal or external consequence for them) and 187 norms (statements describing the behaviour of an actor, which includes internal or external constraints or obligations on its action). None of the investigated EAPs includes rules (an institutional statement describing an actor's behaviour, which includes sanctions if the goal is not respected). Of the 259 institutional statements in the 7th EAP, 44.8% are strategies (116 institutional statements), and 55.2% (143 institutional statements) are norms. Of 54 institutional statements in the 8th EAP, 18.5% are strategies (11), and 81.5% are norms (43).

When analysing the distribution of statement types by priority objectives, it resulted that in the 7th EAP the largest number of strategies and norms belongs to the general principles section and economy-related objectives (Table 5). The lowest number of strategies are included in integration into other policy areas and sustainable cities-related objectives. The lowest number of norms are present in sustainable cities and law enforcement-related chapters (Table 5). In the 8th EAP, the number of strategies and norms are evenly distributed, except for norms for the general principles chapter (Table 6). The 8th EAP has a much larger number of norms compared to the 7th EAP.

**Table 5:** Distribution of strategies and norms by priority objectives in the 7th Environment Action Programme.

Priority objective	Number of strategies	%	Number of norms	%
General principles	25	9.7	26	10.0
O1 Natural capital	15	5.8	17	6.6
O2 Economy	20	7.7	28	10.8
O3 Human health and well-being	12	4.6	8	3.1
O4 Law enforcement	13	5.0	4	1.5
O5 Knowledge	7	2.7	14	5.4
O6 Economy	6	2.3	18	6.9
O7 Integration into other policy areas	3	1.2	8	3.1
O8 Sustainable cities	3	1.2	5	1.9
O9 International dimension	12	4.6	15	5.8

**Table 6:** Distribution of strategies and norms by priority objectives in the 8th Environment Action Programme.

Priority objective/thematic	Number of strategies	%	Number of norms	%
General principles	3	2.08	25	17.4
O1 Greenhouse	4	2.78	15	10.4
O2 Climate change	5	3.47	14	9.7
O3 Growth model	3	2.08	16	11.1
O4 Zero pollution	2	1.39	14	9.7
O5 Biodiversity	6	4.17	16	11.1
O6 Sustainable economy	6	4.17	15	10.4

### 3.3 EAPs enforcement perspectives

When analysing EAPs enforcement perspectives using the deontic included in each institutional statement, it resulted that 55% of the statements in 7th EAP (143 out of 259) include a deontic component (a prescriptive element of the legislation that specifies what is allowed, mandatory, or prohibited) while in the 8th EAP, the proportion increased to 81% (i.e., 43 institutional statements out of 54).

In the 7th EAP, out of the 143 statements with a deontic component, 84 describes allowed actions and 59 mandatory actions. There are no prohibited actions. Of the allowed actions, 13.1% are in the form of norms without an object component (ADIC simple norms), and 19.3% are in the form of norms with an object component (ABDIC complex norms). The most frequent deontic is should (72 statements, 50.3%), followed by can, could, and may (13 statements, 9.1%). In the case of required actions, 12.7% are norms without an object component (ADIC simple norms), and 10% are norms with an object component (ABDIC complex norms). The most frequent deontic is need (35 statements, 24.5%), followed by shall (14 statements, 9.8%), required (9 statements, 6.3%), and must (one statement, 0.7%).

In the 8th EAP, out of 43 statements with the deontic component, 21 describe allowed actions and 23 mandatory actions. There are no prohibited actions. Of the allowed actions, 27.9% are in the form of norms without object components (ADIC simple norms) and 18.6% as norms with object components (ABDIC complex norms). The most frequent and the only deontic is should (20 statements, 46.5%). In the case of required actions, 23.3% are norms without an object component (ADIC simple norms), and 30.2% are norms with an object component (ABDIC complex norms). The most frequent and the only deontic is shall (23 statements, 53.5%).

**Fig. 1:**
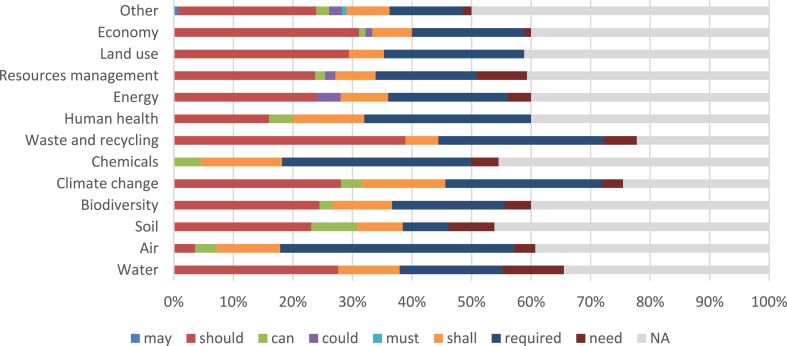
Distribution of deontic verbs by environmental domains (7th EAP).

**Fig. 2:**
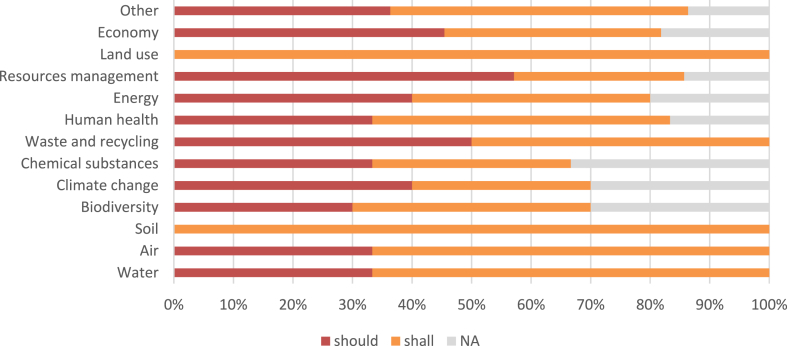
Distribution of deontic verbs by environmental domains (8th EAP).

For a broader characterisation of enforcement perspective, we separated the deontic by environmental domain, i.e., water, air, soil, biodiversity, climate change, chemicals, waste and recycling, human health, energy, resource management, land use, economy and others (e.g., policies, research). In the 7th EAP significantly more required actions are registered for air quality, climate change, biodiversity, chemicals, and human health, while significantly more nonbinding actions are defined for economy and policy and governance (other) domains (Fig. 1). In 8th EAP, except for soil, land use, and policy and governance (other), the EU Commission defined an equal number of required and nonbinding actions (Fig. 2). Resource management is the only domain in which there are more allowed actions than required actions.

### 3.4 Association between stakeholders and type of statements

The results of bipartite networks analysis formed by actors (frameworks/institutions/stakeholders) of each institutional statement (attribute in IGT syntax) with the type of institutional statement indicate that despite the large differences in terms of the number of statements in the two EAPs, the cohesion of the networks is similar. The densities of the two networks are 0.46% (indicating that both networks have the same proportion of potential connections between statements and attributes that are actually present), the two networks are not fragmented (i.e., no actor acts in isolation), and transitivity is 0.7 (i.e., the networks contain groups of statements and attributes that are densely connected internally). Furthermore, the networks have a diameter (the shortest path length from every node to all other nodes) of four, further confirming the structural identity of the two EAPs.

**Fig. 3:**
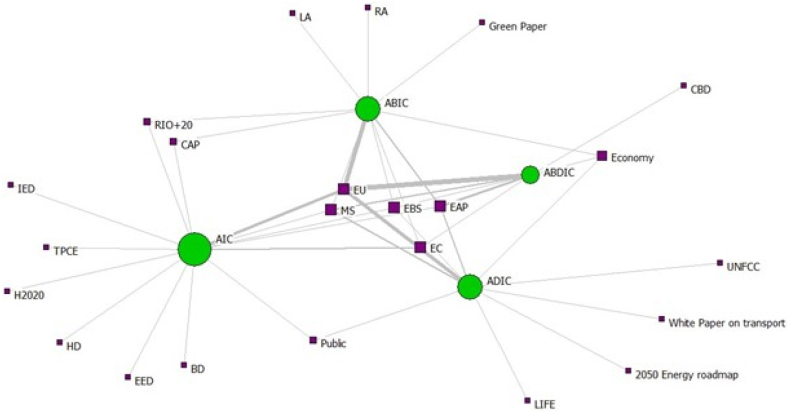
Two-mode network illustrating attributes connected to statement type for the 7th EAP (size of nodes given by normalised degree centrality, node colour given by node type - green statement, purple attribute, link thickness by tie strength).

**Fig. 4:**
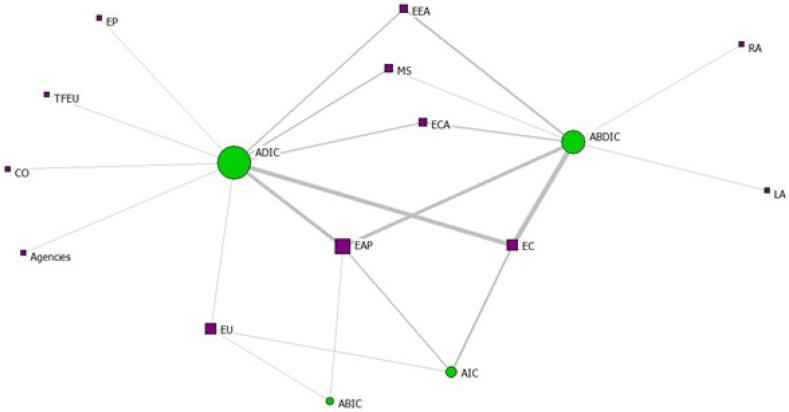
Two-mode network illustrating attributes connected to statement type for the 8th EAP (size of nodes given by normalised degree centrality, node colour given by mode - green statement, purple attribute, link thickness by tie strength).

When analysing the normalised degree centrality results calculated for the types of statement in the two analysed networks, we found that the most central type of statement is AIC (simple strategies) for the 7th EAP (Fig. 3, Table 7), which means that emphasis was placed on a simple linguistic structure. However, in the case of the 8th EAP (Fig. 4, Table 7), the ADIC type (simple norms) represented the most central type, linguistically similar to the 7th EAP, but focused on policy implementation. Furthermore, when analysing the normalised degree centrality of actors (frameworks/institutions/stakeholders) (i.e., who is in charge of the implementation of the statement), EAP itself as a policy framework, European Environment Agency, Member States, and the European Union are the most central in both EAPs (i.e., well positioned for collaborative implementation of the actions indicated in the EAPs).

**Table 7:** Acronyms used in Fig. 3, Fig. 4.

Acronym	Name of actors (frameworks/institutions/stakeholders)
BD	Birds Directive
CAP	Common Agricultural Policy
CBD	Convention on Biological Diversity
CO	The Council of the European Union
EAP	Environment Action Programme
EBS	EU Biodiversity Strategy
EC	European Commission
ECA	European Chemicals Agency
EEA	European Environment Agency
EED	Energy Efficiency Directive
EU	European Union
H2020	Horizon 2020 Programme
IED	Industrial Emissions Directive
LA	Local authorities
LIFE	LIFE Programme
MS	Member States
RA	Regional authorities
RIO+20	RIO +20 Earth Summit 2012
STH	Stakeholders
TFEU	Treaty on the Functioning of the European Union
TPCE	Technical Platform for Cooperation on the Environment

## 4 Discussion

By combining International Grammar Tool and network analysis, we found that the EU 8th Environment Action Programme (2021–2030) is more streamlined and target oriented as compared to 7th Environment Action Programme. For example, apart from a reduced number of objectives and institutional statements, the statements in the 8th EAP include only prescriptive elements (*should* and *shall* as deontic), making the legal text easier to understand, apply, and monitor. Moreover, institutional statements in the 8th EAP are to be implemented mostly at European Union and European Commission level, while in the 7th EAP the number of actors (frameworks/institutions/stakeholders) is higher and often regional or local (e.g., European Union, Environment Action Programme, European Environment Agency, European Commission, European Parliament, Birds Directives, Common Agricultural Policy, The Convention on Biological Diversity, Regional authorities, Local authorities).

The analysis of the 7th and 8th EAPs revealed significant changes in the priority objectives and environmental themes covered. The number of priority objectives decreased from nine to six, and the themes are more closely related to Sustainable Development Goals [[52], [53], [54]]. Furthermore, the formulation of the 8th EAP objectives indicates the desired outcomes and impact more clearly, making them more specific and measurable. This might be because when replacing the 7th EAP, the European Commission saw this opportunity as a step towards achieving a vision for 2050 strongly related to the UN Sustainable Development Goals. Furthermore, the objectives of the 8th EAP fully endorse the environmental and climate objectives of the European Green Deal (EGD) [55], which is itself a document aligned with environmental and climate objectives defined in the 2030 Agenda for Sustainable Development Goals [[56], [57], [58], [59]]. In addition to the EGD and the 2030 Sustainable Development Goals Agenda [60]^,^ the goals of the 8th EAP are tightly interconnected with the Paris Climate Agreement (2015) and the Addis Ababa Action Agenda on Financing for Development [61,62]. For example, the objectives set in the 8th EAP indicate that the EU must advance towards a neutral economy and a good state of the planet and human health [63].

The two EAPs are different not only in terms of priority objectives formulation but also in terms of institutional statements, as shown by IGT analysis. The number of institutional statements decreased by 81% in the 8th EAP compared to the 7th EAP, and the legislators clearly favoured norms-like statements instead of strategies-like statements (see Table 5, Table 6). This feature suggests that the European Commission is more likely to develop implementable programmes than less enforceable strategies [64]. EAP norms outnumber strategies-like statements in all priority objectives of 8th EAPs but only for Natural capital, Economy, Knowledge, Integration and EU international dimension in 7th EAP (see Table 5, Table 6). This reinforces the target-oriented characteristic of the 8th EAP. A similar pattern of movement toward more actionable legal documents was also observed by Siddiki et al. [41] when comparing several Colorado State legal documents (Colorado Aquaculture Act, CAA Administration and Enforcement Rules, Article VII of the Chapter 00 Regulations and the Fish Health Board Statute). Kamran and Shivakoti [65] analysed the legal documents of irrigation systems in the Punjab region of Pakistan and inferred that externally developed rules failed to fully incorporate the local needs and were too complex to apply. They also concluded that institutional grammar tool needs wider applicability in different linguistic contexts (e.g., in languages other than English). This highlights the importance of streamlining of legal documents, an objective achievable when using IGT before legally binding the norms.

IGT allows identifying the type of statement and enforcement perspectives using the deontic verbs included in each statement [66]. The enforcement perspectives enabled by IGT are important because they allow us to understand the power of environmental policies [29] and what are the most important domains in the respective document. The 7th EAP includes many deontic modals in statements, such as should, can, could, may, shall, need, and required. In the 8th EAP the wording is simplified, EU legislators using only shall and should. While in the 7th EAP required actions are described with a greater frequency for air quality, climate change, biodiversity, chemical and human health, in the 8th EAP the legislators choose to include an equal proportion of required/nonbinding actions (see Fig. 1, Fig. 2). The 7th EAP presents a much greater linguistic complexity and provides a large volume of information but fewer tools to achieve the priority objectives. Furthermore, the 8th EAP is focused on intentions and obligations, both from the perspective of achieving EAP priority objectives and the overarching European Green Deal [36].

When analysing the network formed between stakeholders (institutions, frameworks, stakeholders) and the type of statements in each EAP, the density of the two graphs illustrates that both analysed EAPs have an equal number of ties between the statements and attributes, despite the differences in the number of statements, emphasising the absence of improvements in collaboration for implementing environmental policies [67,68]. For the 7th EAP, the degree centrality results show high scores for simple strategies, European Union, EU Biodiversity Strategy, Member States, Environment Action Programme and European Commission, while for the 8th EAP, the highest degree centralities are obtained for simple norms, Environment Action Programme, European Environment Agency, European Chemicals Agency. Usually, the highest degree centrality indicates the most popular nodes in the network or, in our case, stakeholders, legal norms, institutions or statements types crucial for environmental policy implementation in the context of Environment Action Programmes [69,70].

Our results show that the linguistic style of Environmental Action Programmes is important for implementing and monitoring environmental policies in the European Union. By analysing the two documents, it was possible to determine the areas addressed more thoroughly in the two EAPs and the main actors included in the linguistic constructions. Furthermore, we showed that the most recent two EAPs are closely linked and constructed to take into account and influence other EU and international policies. This is more evident in the 8th EAP. The findings of this study on the links between the 7th EAP and the 8th EAP provide the basis for future research on the integration of environmental policies into other strategic goals of the European Union. The close links of the 8th EAP with the 2030 for Sustainable Development Goals and the European Green Deal represented an important step toward the internationalisation of the EU environmental agenda and integration of other policies into environmental policies. Our research findings can support actors who must or should fulfil the priority objectives set in the current Environment Action Programme (8th EAP). Although it is a useful tool for analysing political content, the coding process is lengthy and relies on the coders' expertise, especially when analysing other than English documents. However, the Institutional Grammar Tool is a method that helps obtain comparable results and can be used to improve the legal process before legally binding documents and after assessing the documents in force and suggest improvements for the next generation of policy documents. Future research should assess and analyse the link between the content of environmental policy documents and their implementation in the field to better document the gap between theory and practice in environmental governance.

## 5 Conclusions

This study uses Institutional Grammar Tool and network analysis to document the evolution of the last two European Environment Action Programmes, the 7th EAP and 8th EAP. IGT helps us identify changes in environmental policy by analysing the legislative content of statements in EAPs. The analysis of the statements aims to identify the evolutionary characteristics from the perspective of priority objectives, domains, implementation, and types of actions.

Following the documentation on the priority objectives of the two programmes, it was noticed that from a quantitative point of view, the 8th EAP has fewer objectives compared to the 7th EAP, being built following the 2030 Agenda for Sustainable Development and the European Green Deal.

Using IGT, it was possible to understand the changes in the structure of European environmental governance by classifying the statements from the two EAPs. Therefore, it was concluded that the 7th EAP presents a higher number of institutional statements, with a higher degree of centrality in the case of simple strategies, with a higher grammatical complexity, especially in the case of the deontic component, a balanced number of types of statements (strategies and norms), and with several required actions, especially in the domains such as air quality, climate change, biodiversity, chemical and human health (see Fig. 1). The 8th EAP is more compressed and systematic, with the degree of centrality higher in the case of simple norms, focused mostly on norm-type declarations, and the type of actions (required/permitted) are distributed proportionally in the case of all domains (see Fig. 2).

In conclusion, there is an advancement from the 7th EAP to the 8th EAP, showing that EU environmental policies are continuously evolving and improving for a better implementation and applicability in real life environmental protection and sustainability.

## Author contribution statement

Conceived and designed the experiments: LCP and LR; Performed the experiments: LCP and LR; Analysed and interpreted the data: LCP, LR, AN; Contributed reagents, materials, analysis tools or data: LCP, LR, AN, IMN, SM; Wrote the paper: LCP, LR, AN, IMN, SM. LCP and LR contributed equally to this work.

## Funding

IMN work was supported by a grant from the 10.13039/100018987Romanian Ministry of Research, Innovation, and Digitisation, 10.13039/100003950CNCS - 10.13039/100010587UEFISCDI, project number PN–III–P1-1.1-TE-2021-1067, within PNCDI III. LCP work was funded by the 10.13039/501100007336University of Bucharest, Faculty of Geography, 10.13039/100009138Doctoral School Simion Mehedinti Nature and Sustainable Development.

## Data availability statement

Data will be made available on request.

## Declaration of competing interest

The authors declare that there is no conflict of interest.
